# Gonadotropins and Sex Steroid Hormones in Captive-Reared Small Yellow Croaker (*Larimichthys polyactis*) and Their Role in Female Reproductive Dysfunction

**DOI:** 10.3390/ijms24108919

**Published:** 2023-05-17

**Authors:** Kang Hee Kho, Zahid Parvez Sukhan, Seok-Woo Yang, Nam-Yong Hwang, Won-Kyo Lee

**Affiliations:** 1 Department of Fisheries Science, Chonnam National University, Yeosu 59626, Republic of Korea; kkh@chonnam.ac.kr (K.H.K.); sukhan1026@jnu.ac.kr (Z.P.S.); 2 Ocean and Fisheries Science Institute, Jeollanam-do 59326, Republic of Korea; ysw0219@korea.kr (S.-W.Y.); hny2946@korea.kr (N.-Y.H.)

**Keywords:** Gonadotropins (GtH), steroids, reproduction, reproductive dysfunction, small yellow croaker, *Larimichthys polyactis*

## Abstract

The seed production of small yellow croaker (SYC) is constrained by reproductive dysfunction in captive-reared females. Reproductive dysfunction is closely linked to endocrine reproductive mechanisms. To better understand the reproductive dysfunction in captive broodstock, functional characterization of gonadotropins (GtHs: follicle stimulating hormone β subunit, *fshβ;* luteinizing hormone β subunit, *lhβ;* and glycoprotein α subunit, *gpα*) and sex steroids (17β-estradiol, E2; testosterone, T; progesterone; P) was performed using qRT-PCR, ELISA, in vivo, and in-vitro assay. The pituitary GtHs and gonadal steroids levels were significantly higher in ripen fish of both sexes. However, changes in *lhβ* and E2 levels in females were not significant in the developing and ripen stages. Furthermore, GtHs and steroids levels were lower in females compared to males throughout the reproductive cycle. In vivo administration of gonadotropin releasing hormone analogue (GnRHa) significantly increased the expression of GtHs in both dose- and time-related manners. The lower and higher doses of GnRHa led to successful spawning in male and female SYC, respectively. Sex steroids in vitro significantly inhibited the expression of *lhβ* in female SYC. Overall, GtHs were shown to play a vital role in final gonadal maturation, while steroids promoted negative feedback in the regulation of pituitary GtHs. Lower levels of GtHs and steroids might be key components in the reproductive dysfunction of captive-reared female SYC.

## 1. Introduction

The reproduction of teleost fish is regulated by a complex process in the brain–pituitary–gonad (BPG) axis. The reproductive developmental process involves a complex interaction between the gonadotropin-releasing hormone (GnRH) in the brain, gonadotropins (GtHs) in the pituitary gland, sex steroid hormones in the gonads, and other neurohormones. GtHs are synthesized and secreted from the pituitary gland, which stimulates the gonads to produce sex steroids [1]. During this process, the brain GnRH acts as an upstream regulator of pituitary GtHs [2,3]. Generally, two forms of GtHs are secreted from the pituitary gland: the follicle-stimulating hormone (FSH) and the luteinizing hormone (LH). These GtHs contain a common glycoprotein α subunit (*gpα*) that forms a heterodimer with a hormone-specific β subunit (follicle stimulating hormone β subunits, *fshβ*; and luteinizing hormone β subunit, *lhβ*) [4,5]. GtHs plays a vital role in the endocrine control of reproduction and the gonadal maturation process in fish [2,4]. LH is believed to stimulate the gonads to synthesize and secrete steroid hormones. However, FSH plays a pivotal role in both vitellogenesis and spermatogenesis, whereby it regulates the ovarian and testicular development processes in fish [6]. Several studies have reported the role of GtHs in the regulation of gametogenesis and final gonadal maturation in fish, including Nile perch [7], jack mackerel [8], chub mackerel [9], Mediterranean seabass [10], and red seabream [11].

Furthermore, sex steroid hormones also play a crucial role in the gametogenesis and gonadal maturation process of fish [12]. Steroid hormones, such as 17β-estradiol (E2), testosterone (T), and progesterone (P), are produced in the gonads with the stimulatory effect of pituitary GtHs. In fish, E2 and T have been identified during gonadal development, while P has been identified during ovarian and testicular maturation [13]. Furthermore, E2, which is produced in ovarian follicles, is important for ovarian development in fish. GtHs stimulate the secretion of sex steroids from the granulosa cells of developing and mature oocytes, which proceed to regulate vitellogenesis and final oocyte maturation [14]. Changes in gonadal and serum levels of steroid hormones during the reproductive cycle are reported in several fish species, including Nile perch [7], blue tang [15], Southern hake [16], European seabass [17], Caspian kutum [18], Korean spotted seabass [19], and tilapia [20].

It is extremely important for the reproductive endocrine system to function at an optimal level in order to complete gonadal development and maturation, and for successful spawning. However, most captive-reared fish show a degree of reproductive dysfunction, specifically female fish. Generally, three kinds of reproductive dysfunction are observed in captive-reared fishes: (a) vitellogenesis and spermatogenesis of fish undergo a complete failure, (b) absence of final oocyte maturation, and (c) absence of spawning at the end of the reproductive cycle [21]. All these reproductive dysfunctions typically arise from the combination of captivity-induced stress and the lack of an appropriate natural spawning environment, which ultimately leads to endocrinological dysfunction [2]. Endocrinological dysfunction, in relation to GtHs and sex steroids in captive female fishes, has been studied in various fishes, including jack mackerel [8], yellowtail tetra [22], golden mahseer [23], mahi-mahi [24], and sablefish [25]. To overcome the problems of reproductive dysfunction in captive broodstock, it is imperative to study the detailed endocrinological function and mechanisms underlying reproductive dysfunction in a particular captive-reared fish species. Ultimately, this process will help to develop strategies that optimize the production of fish in captivity.

The small yellow croaker (SYC), *Larimichthys polyactis*, is a marine demersal fish belonging to the order Perciformes. SYC are widely distributed on the coast of the Korean Peninsula, the Bohai Sea, and the East China Sea [26]. It is an oceanodromous fish that recurrently migrates among feeding, overwintering, and spawning grounds [27,28]. SYC are one of the most commercially important fish in the Republic of Korea, Japan, and China [26]. It is a popular fish in the Republic of Korea for its high nutritional value and medicinal uses. It has been utilized as a food resource for a long time in the Republic of Korea [29]. However, the wild population of SYC has been harshly depleted due to overfishing and the destruction of its natural habitat [30]. Indeed, farming of SYC has been practiced in Southeast Asian countries, including the Republic of Korea, using wild seeds. Recently, artificial breeding of SYC has been developed using captive-reared broodstock in China and the Republic of Korea [31]. However, female broodstock of SYC raised in captivity failed to undergo final gonadal maturation and spawning [32,33]. Information on the reproductive development and breeding of SYC has been reported in several studies [34,35,36]. Moreover, a recent study reported that *gnrh1* has a possible role in the reproductive dysfunction of captive-reared female SYC [32]. However, reproductive endocrinological studies associated with GtHs and sex steroids have not yet been published. To improve the understanding of the reproductive endocrine system, related to GtHs and sex steroids in SYC and their possible role in reproductive dysfunction in captive-reared females, several experiments have been performed in this study. First, mRNA expression levels of GtH subunits in the pituitary were analyzed and the levels of sex steroids in serum and gonads were observed at different reproductive stages. Then, the effects of GnRHa on pituitary GtHs were observed in vivo. Finally, an assay evaluating the feedback regulation of the sex steroids (E2 and MT) on pituitary GtHs was performed in vitro.

## 2. Results

### 2.1. Distribution of fshβ, lhβ, and gpα in Different Organs of Captive-Reared Mature SYC

**Figure 1:**
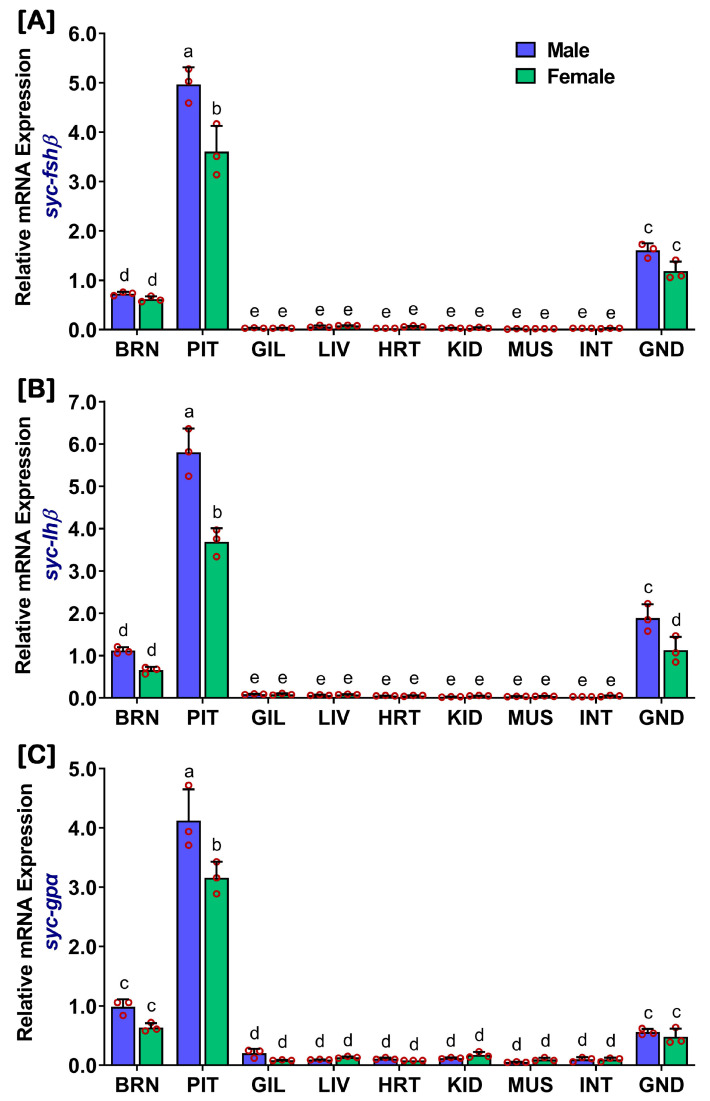
Relative mRNA expression levels of *fshβ*, *lhβ*, and *gpα* subunits in different organs of captive-reared mature male and female SYC. (**A**) *fshβ*, (**B**) *lhβ*, and (**C**) *gpα*. The mRNA expression data were normalized to *β-actin*. Raw data points (red circles) in the bar graphs represent biological replicates, different letters above the bars indicate significant differences (*p* < 0.05) among different organs, and error bars represent the standard error of the mean (SEM). BRN, brain; PIT, pituitary; GIL, gill; LIV, liver; HRT, heart; KID, kidney; MUS, muscle; INT, intestine; GND, gonad.

The mRNA expression levels were examined in different organs of captive-reared mature SYC, and this revealed that *fshβ* (Figure 1A), *lhβ* (Figure 1B), and *gpα* (Figure 1C) were predominantly expressed in the pituitary. However, captive-reared mature females expressed significantly lower levels of all three GtHs compared to mature males (Supplementary Table S1). Furthermore, all three GtH subunits were expressed in the brain and gonads of both sexes, which were at significantly lower levels than in the pituitary.

### 2.2. Gonadal Histology

**Figure 2:**
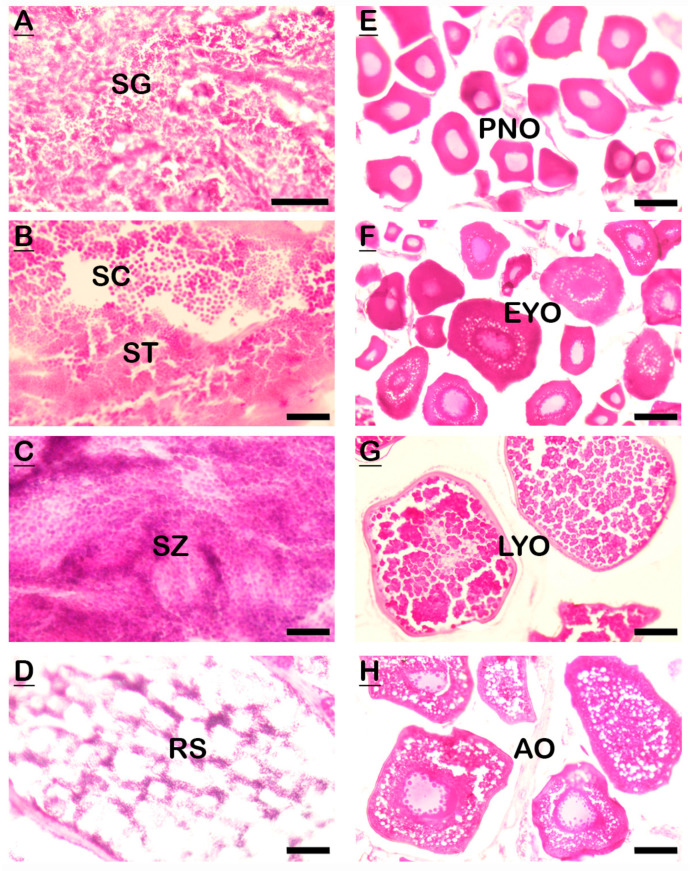
Photomicrograph showing gonadal developmental stages of male (**A**–**D**) and female (**E**–**H**) SYC. (**A**,**E**) Immature stage (IM). (**B**,**F**) Developing stage (DS). (**C**,**G**) Ripen stage (RS). (**D**,**H**) Spent stage (SS). SG, spermatogonia; SC, spermatocyte; ST, spermatid; SZ, spermatozoa; RS, residual spermatozoa; PNO, peri-nucleolar oocyte; EYC, early yolk oocyte; LY, late yolk oocyte; AO, atretic oocyte. Scale bars = 100 µm.

The representative gonadal tissue sections of different developmental stages of male (Figure 2A–D) and female (Figure 2E–H) SYC are presented in Figure 2. Gonad samples during the start of experiment were at the immature (IM) stage. With the progress of the experiment, gonadal development was observed and identified as the developing stage (DS), ripen stage (RS), and spent stage (SS). IM testis contained cysts of spermatogonia (Figure 2A), DS testis were characterized by the spermatocytes and spermatids (Figure 2B), RS testis were filled with spermatozoa (Figure 2C), and at this stage milt was obtained by gentle abdominal pressure, while SS testis contained only residual spermatozoa (Figure 2D). However, IM ovaries were characterized by peri-nucleolar oocytes (Figure 2E), DS ovaries contained early yolk oocytes (Figure 2F), RS ovaries showed late yolk oocytes (Figure 2G), and SS ovaries were characterized with atretic oocytes (Figure 2H).

### 2.3. Changes in mRNA Expression Levels of fshβ, lhβ, and gpα in the Pituitary at Different Gonadal Developmental Stages

**Figure 3:**
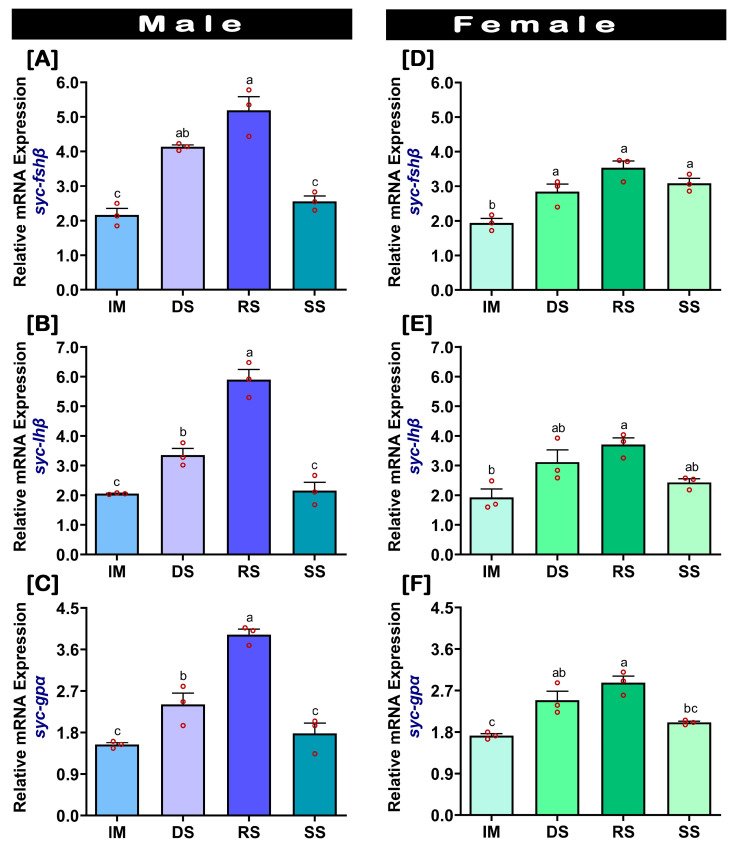
Relative mRNA expression levels of *fshβ*, *lhβ*, and *gpα* subunits in the pituitary during gonadal developmental stages in captive-reared male and female SYC. (**A**,**D**) *fshβ*, (**B**,**E**) *lhβ*, and (**C**,**F**) *gpα*. The mRNA expression data were normalized to *β-actin*. Raw data points (red circles) in all the bar graphs represent biological replicates, different letters above the bars indicate significant differences (*p* < 0.05) among developmental stages, and error bars represent the standard error of the mean (SEM). IM, immature; DS, developing stage; RS, ripen stage; SS: spent stage.

Temporal changes in the expression levels of *fshβ*, *lhβ*, and *gpα* in the pituitary of captive-reared SYC during gonadal development stages are presented in Figure 3. The mRNA expression levels of *fshβ*, *lhβ*, and *gpα* were higher in males during the RS stage (Figure 3A–C) compared to the other stages. Contrastingly, the mRNA expression levels of *fshβ* and *lhβ* did not show any significant changes among DS, RS, and SS in females (Figure 3D,E). However, the expression levels of *fshβ*, *lhβ*, and *gpα* were significantly higher in RS compared to IM (Figure 3D–F). Furthermore, significantly lower levels of *fshβ*, *lhβ*, and *gpα* expressions were observed in the RS of the captive-reared females compared to males (Supplementary Table S2).

### 2.4. In Vivo Effect of GnRHa on mRNA Expression of fshβ, lhβ, and gpα in Pituitary

**Figure 4:**
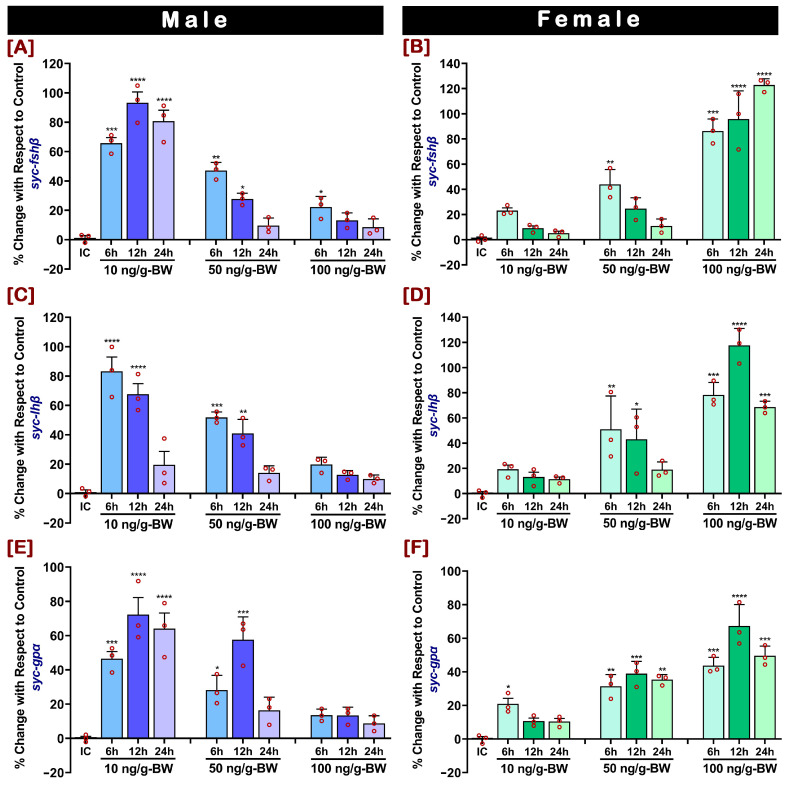
Dose- and time-related in vivo effects of GnRHa on pituitary *fshβ*, *lhβ*, and *gpα* mRNA levels in male and female SYC. (**A**,**B**) *fshβ*, (**C**,**D**) *lhβ*, and (**E**,**F**) *gpα*. GnRHa was administered intramuscularly at 10, 50, and 100 ng/g-BW concentrations and treated for 6, 12, or 24 h. The mRNA expression data were normalized to *β-actin*. Expression results (mean ± SEM) are presented as percentage changes against the initial control (IC), raw data points (red circles) in all the bar graphs represent biological replicates and asterisks above the bars indicate significant differences (*p* < 0.05) compared to the zero time point initial control.

The in vivo administration of GnRHa promoted both dose- and time-related differential expressions of *fshβ*, *lhβ*, and *gpα* in the male and female pituitary glands. The percentage changes in the mRNA expressions of *fshβ*, *lhβ*, and *gpα* in males were significantly increased at the lower dose of GnRHa (Figure 4A,C,E), whereas it was significantly increased at the higher dose of GnRHa in females (Figure 4B,D,F). In males, the percentage changes in the mRNA expressions of *fshβ* and *gpα* were significantly higher at 12 h post-injection; however, percentage changes in *lhβ* were significantly higher at 6 h post-injection after the lower doses. Conversely, in females, the percentage change in mRNA expression of *fshβ* was significantly higher at 24 h post-injection, and the changes in *lhβ* and *gpα* were significantly higher at 12 h post-injection. Additionally, the higher dose of GnRHa in males and the lower dose of GnRHa in females did not show any significant changes in the expression of any GtH subunits.

### 2.5. Changes in mRNA Expression Levels of fshβ, lhβ, and gpα in the Pituitary at Different Induced Spawning Events

**Figure 5:**
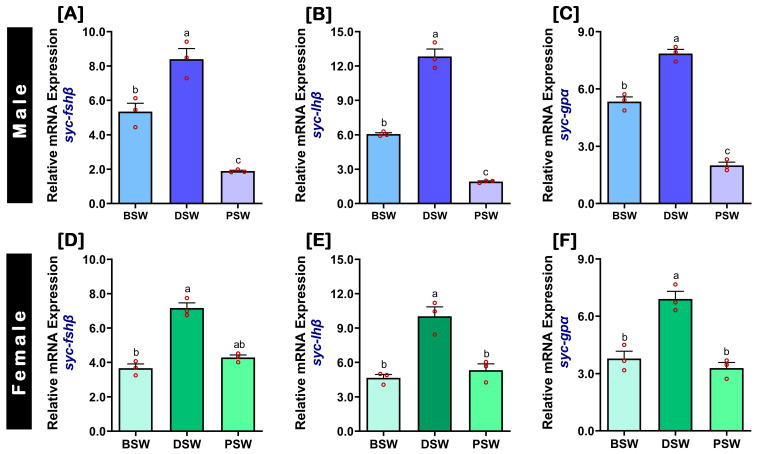
Relative mRNA expression levels of *fshβ*, *lhβ*, and *gpα* subunits in the pituitary during induced spawning events in captive-reared male and female SYC, induced with GnRHa. (**A**,**D**) *fshβ*, (**B**,**E**) *lhβ*, and (**C**,**F**) *gpα*. The mRNA expression data were normalized to *β-actin*. Raw data points (red circles) in all bar graphs represent biological replicates, different letters above the bars indicate significant differences (*p* < 0.05) among spawning events, and error bars represent the standard error of the mean (SEM). BSW, before spawning; DSW, during spawning; PSW, post-spawning.

Following induction with GnRHa, the pituitary levels of *fshβ*, *lhβ*, and *gpα* mRNA expressions in male (Figure 5A–C) and female (Figure 5D–F) SYC were significantly higher at during spawning (DSW) stage than the two other stages during induced spawning. However, the pituitary levels of *fshβ*, *lhβ*, and *gpα* mRNA expressions in females were significantly lower at DSW stage compared to males (Supplementary Table S3).

### 2.6. Changes in Gonadal and Serum Levels of Sex Steroids at Different Gonadal Developmental Stages of Captive-Reared SYC

**Figure 6:**
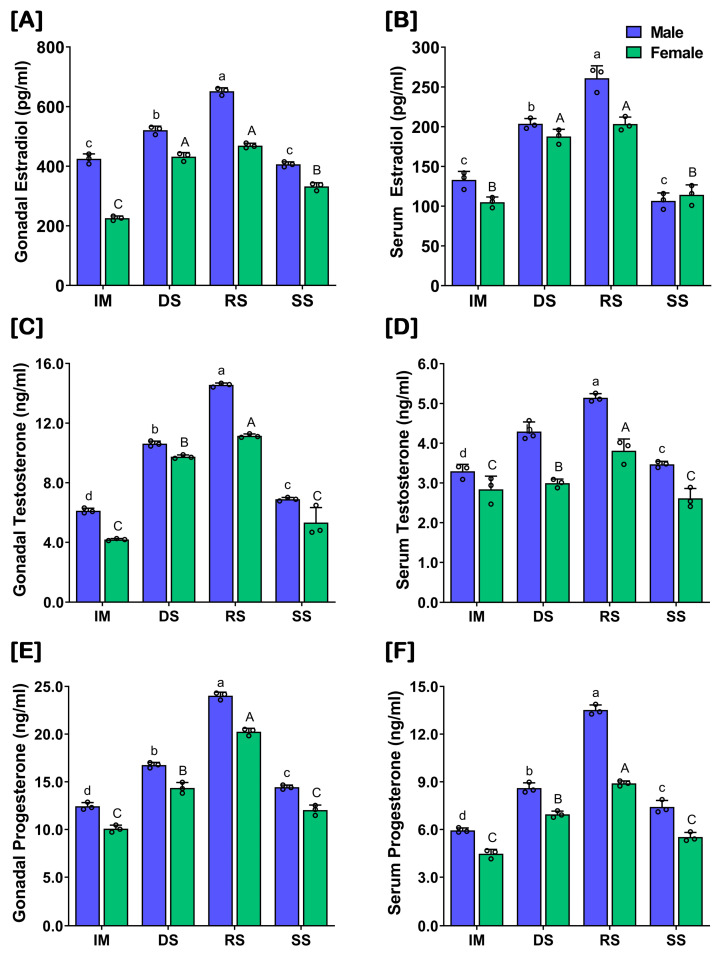
Level of sex steroids in the gonads and serum of captive-reared male and female SYC during gonadal developmental stages. (**A**,**B**) estradiol (E2), (**C**,**D**) testosterone (T), and (**E**,**F**) progesterone (P). Raw data points (black circles) in all bar graphs represent biological replicates, different letters (lower-case letter for male and upper-case letter for female) above the bars indicate significant differences (*p* < 0.05) among developmental stages, and error bars represent the standard error of the mean (SEM). IM, immature; DS, developing stage; RS, ripen stage; SS, spent stage.

The levels of the sex steroids, E2, T, and P in the gonads and serum were significantly higher at RS in captive-reared male and female SYC during the gonadal developmental stages (Figure 6). However, the levels of these sex steroids were differentially expressed in the captive-reared male and female SYC, whereby the females showed a significantly lower expression compared to the males (Supplementary Tables S4 and S5). However, there was no significant increase in E2 levels at RS compared to DS in females (Figure 6B). The levels of the sex steroids in the gonads (Figure 6A,C,E) and serum (Figure 6B,D,F) showed similar patterns. However, the concentration of each sex steroid was lower in the serum compared to the gonads in both sexes.

### 2.7. In Vitro Effect of 17β-Estradiol (E2) on fshβ and lhβ mRNA Levels in Cultured Pituitary

**Figure 7:**
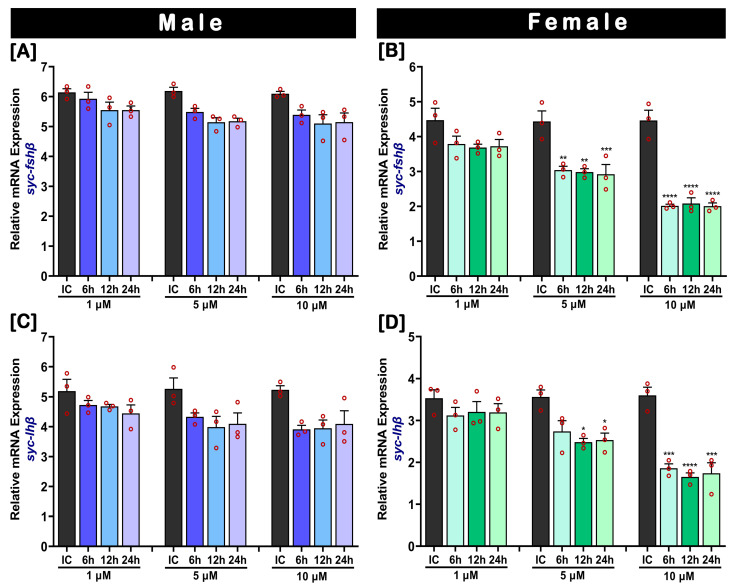
Dose- and time-related in vitro effects of 17-β estradiol (E2) on *fshβ* and *lhβ* mRNA levels in cultured pituitaries of SYCs (**A**,**B**) *fshβ* and (**C**,**D**) *lhβ*. Pituitaries were cultured in a culture media with E2 concentrations of 1, 5, and 10 µM and treated for 6, 12, or 24 h. The mRNA expression data were normalized to *β-actin*. Raw data points (red circles) in all bar graphs represent biological replicates, different asterisks above the bars indicate significant differences (*p* < 0.05) compared to the zero time point initial control (IC), and error bars represent the standard error of the mean (SEM).

The in vitro effect of E2 on *fshβ* and *lhβ* mRNA expression in the cultured pituitary of male and female SYC is presented in Figure 7. The results of the in vitro assay revealed that E2 significantly inhibited *fshβ* and *lhβ* mRNA expression in the female pituitary, in a dose-related manner. A higher dose (10 µM) of E2 significantly inhibited the expression of *fshβ* and *lhβ* (Figure 7B,D). However, no significant effect of E2 was observed on the mRNA expressions of *fshβ* and *lhβ* compared to the initial control at a lower dose (1 µM). In males, no dose- or time-related effects relating to E2 were observed in the expression of *fshβ* and *lhβ* (Figure 7A,C).

### 2.8. In Vitro Effect of 17α-Methyltestosterone (MT) on fshβ and lhβ Levels in Cultured Pituitary

**Figure 8:**
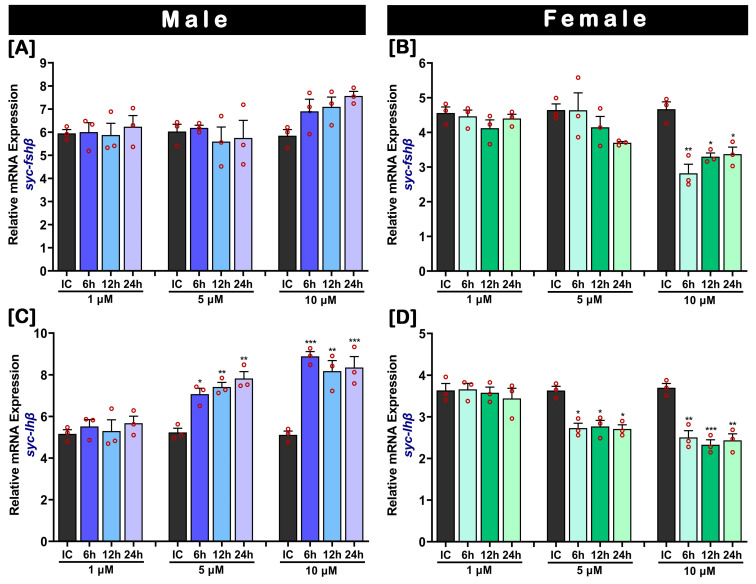
Dose- and time-related in vitro effects of 17-α methyltestosterone (MT) on *fshβ* and *lhβ* mRNA levels in cultured pituitaries of SYC. (**A**,**B**) *fshβ*, and (**C**,**D**) *lhβ*. Pituitaries were cultured in a culture media with MT concentrations of 1, 5, and 10 µM and treated for 6, 12, or 24 h. The mRNA expression data were normalized to *β-actin*. Raw data points (red circles) in all bar graphs represent biological replicates, different asterisks above the bars indicate significant differences (*p* < 0.05) compared to the zero time point initial control (IC), and error bars represent the standard error of the mean (SEM).

The dose- and time-related in vitro effects of MT on pituitary *fshβ* and *lhβ* mRNA levels in male and female SYC are illustrated in Figure 8. As observed for the in vitro effect of E2, MT also showed a dose-related inhibitory effect on the mRNA expression levels of *fshβ* and *lhβ* in females. Both medium (5 µM) and higher (10 µM) doses of MT significantly inhibited the expression of *lhβ* at 6 h, 12h, and 24 h compared to the initial control (Figure 8D). The expression of *fshβ* was significantly inhibited by the higher dose only (Figure 8B). However, the lower dose (1 µM) of MT did not imply any changes in mRNA expression levels of *fshβ* and *lhβ* compared to the initial control. Moreover, the *fshβ* expression did not show any significant changes in males, (Figure 8A); however, the expression levels of *lhβ* in males were significantly upregulated in both the medium (5 µM) and higher (10 µM) MT doses (Figure 8C). In the medium dose (5 µM) of MT, the expression of *lhβ* gradually increased from 6 h to 24 h; however, a higher expression was observed after the higher dose (10 µM) at 6 h.

## 3. Discussions

The aim of the present study was to evaluate the regulation of reproductive development, maturation, and spawning of SYC through gonadotropins and sex steroids. Pituitary gonadotropins alongside plasma and gonadal sex steroids play a vital role in the regulation of gonadal maturation and the spawning of fish [2,6]. In the present study, the highest mRNA levels of all three GtH subunits were found in the pituitary of captive-reared SYC, as expected. Additionally, three GtH subunits were expressed in the brain and gonads. GtH subunits were also expressed in very minute amounts in all tested tissues; however, the expression levels of *gpα* in the gill, kidney, and heart were minimally higher than the two other GtH subunits. Similar expression patterns of the GtH subunits were also described in several fishes, including zebrafish [37], tongue sole [38], and rock bream [39]. Furthermore, extrapituitary existence of GtH subunits has also been previously reported in gilthead seabream [40], Atlantic cod [41], and cichlid [42]. The present results align with previous studies, which suggested that extrapituitary expression of GtHs is a common phenomenon in fish. Although the function of pituitary GtHs is well-documented, the function of the extrapituitary GtHs remains unclear [43]. Furthermore, the present study revealed that the expression of the pituitary *fshβ*, *lhβ*, and *gpα* subunits was significantly lower in captive-reared female SYC compared to males. In salmon, it was shown that precious males possessed higher levels of GtHs compared to female parrs, which failed to undergo sexual development [44]. Cultured chub mackerel females also showed significantly lower levels of GtHs compared to males [9]. Similar results were reported in a few other fishes, where captive-reared individuals exhibited lower expressions compared to wild species, including the jack mackerel [8] and rainbow trout [11]. Furthermore, a few studies reported that wild fishes showed similar expression levels between females and males, including the ayu [45].

Gonadal developmental stage-dependent expression analysis of GtH subunits in pituitaries of captive-reared males demonstrated that the expression levels of all three GtH subunits gradually increased from IM to DS to RS, and decreased at SS. The *fshβ* subunit increased at DS and remain statistically similar at RS, whereas the *lhβ* subunit increased at DS and peaked at RS. These results could suggest that *fshβ* initiates testicular development, while *lhβ* is involved in the final maturation at RS. In contrast, the captive-reared female SYC presented a differential expression profile compared to the males, whereby the expression of the GtH subunits increased significantly from IM to DS, and remained unchanged at RS and SS. This result may suggest that the increase in *fshβ* at DS initiates ovarian development. However, a non-significant increase in the GtH subunits, specifically *lhβ* at RS, may suggest that it could not facilitate the final ovarian maturation, which might be the possible reason for ovarian dysfunction in female SYC. Similar expression patterns of the GtH subunits as in the captive-reared female SYC were also reported in several fishes that demonstrated reproductive dysfunctions, including jack mackerel [8], yellowtail tetra [22], mahi-mahi [24], and sablefish [25]. Furthermore, captive-reared female SYC showed lower levels of GtHs compared to males at the DS and RS stages, i.e., female SYC could not synthesize optimal level of GtHs during RS. A similar result was also reported in cultured chub mackerel [9]. Brain *gnrh1* is believed to regulate the secretion of GtHs in the pituitary of fishes [2]. A previous study reported that expression of *gnrh1* in captive-reared SYC also showed lower expression in females during the RS compared to males [32]. Therefore, lower levels of brain *gnrh1* could be the factor that inhibited the secretion and expressions of the pituitary GtH subunits in captive-reared female SYC and led to reproductive dysfunction.

Gamete production is vital for successful aquaculture. However, most captive-reared female fishes reveal some extent of reproductive dysfunction. The most common reproductive dysfunction is the inhibition of the final oocyte maturation [21]. Failure in the final oocyte maturation or ovulation is believed to be caused by decreased stimulation from pituitary *lh*, which occurs through a decline in stimulation from the brain *gnrh* [46]. Several studies reported that administering maturation hormones, especially human chorionic gonadotropin (hCG) and GnRHa, to captive-reared fish could stimulate the release of pituitary GtHs that promote the final gonadal maturation and induce spawning [21,46,47,48]. Induction of GnRHa stimulates the secretion of LH in the pituitary that elevated gonadal and serum LH levels and initiated the final gonadal maturation and spawning [49]. This process ultimately resolved the reproductive dysfunction in captive-reared fishes. However, successful spawning largely depends on a specific dose of GnRHa, which is also species-specific. Hence, it is essential to determine the ideal or optimum dose of GnRHa administration during induced breeding. In the present study, it was observed that in vivo administration of GnRHa significantly changed the expression percentages of GtH subunits, in a dose- and time-related manner, in male and female SYC. Dose- and time-related effects of GnRHa administration on GtHs have also been reported in spotted scat [47], pompano [50], striped bass [51], black porgy [52], Japanese seabass [53], and goldfish [54]. It was found that a lower dose of GnRHa stimulated GtH subunits in male SYC and a higher dose of GnRHa stimulated GtH subunits in female SYC. This result suggests that lower and higher doses of GnRHa are effective for male and female SYC, respectively, during induced spawning.

Next, induced spawning was performed by administering GnRHa with low (10 ng/g-bw) and high (100 ng/g-bw) doses in captive-reared male and female SYC, respectively. Induced spawning with GnRHa promoted the SYC to successfully spawn. Thus, it was observed that the induction of GnRHa during the induced spawning the levels of all three GtH subunits was significantly increased at the spawning stage in both male and female SYC. Finally, the GtH expressions were significantly downregulated in the post-spawning period. This increase in the GtH subunits during the spawning stage may lead to the final gonadal maturation, which induces the onset of spawning. Induction of GnRHa resulted in the increased expression of the GtH subunits in many fishes, including sockeye salmon [6], coho salmon [55], goldfish [56], and Senegalese sole [57]. Moreover, higher mRNA levels for the three GtH subunits were observed in grass puffer during the spawning stage compared to post-spawning season puffer in the wild [58]. A previous study suggested that low levels of brain *gnrh1* in female SYC is a possible reason for reproductive dysfunction in female SYC. It was also shown that the induction of GnRHa during the induced spawning significantly increased the levels of *gnrh1* in female SYC and stimulated the final oocyte maturation and spawning [32]. It is verified that *gnrh1* has been significantly alter the mRNA expression of the GtH subunits in fish [5].

Sex steroid hormones (E2, T, P) also play a vital role in the process of gametogenesis and reproduction. It was shown that the GtH subunits controlled the production of the sex steroid hormones in the gonads of fish [6]. In the present study, the levels of gonadal and plasma E2, T, and P were found to be higher in the RS of captive-reared SYC for both sexes. However, the levels of the sex steroids in female captive-reared SYC were significantly lower compared to males. Several studies reported that captive-reared female fishes contained lower levels of steroid hormones compared to wild and/or captive-reared females and males, including golden sable fish [25], jack mackerel [8], mahi-mahi [24], mahseer [23], and yellowtail tetra [22]. Moreover, those studies also reported that low levels of sex steroids are the possible cause of reproductive dysfunction in captive-reared female fishes, which is related to the findings in the present study.

Alternatively, sex steroid levels eventually control the activity of the BPG axis by binding with sex steroid hormone receptors to induce negative or positive feedback, thus, regulating further hypothalamic and pituitary secretion [59,60]. The feedback regulation of the sex steroids on the hypothalamic GnRH and pituitary GtHs in the BPG axis of mammals and fishes has been studied extensively. In the present study, the expression of *lhβ* in the cultured pituitary of female SYC was significantly downregulated by the in vitro effect of E2 and MT, in a dose-related manner. However, MT presented positive feedback on the *lhβ* expression and E2 did not affect the expression of the GtH subunits in males. The present results revealed that E2 and MT might have a negative feedback role on *lhβ* in female SYC. A previous study reported that E2 and MT have a negative feedback role on the brain *gnrh1*, which is responsible for the release of pituitary GtHs in SYC [32]. Cumulatively, the present and previous results reveal that the sex steroids may regulate the synthesis and release of GtH subunits in SYC, via the action of *gnrh1*. Complex feedback of gonadal sex steroids tightly regulates *gnrh* receptor expressions, thereby affecting the secretion and expression of pituitary GtHs. E2 is potentially the most potent regulator of GnRH and GtH secretion and is generally considered to decrease the expressions of *fshβ* and *lhβ* [61]. The negative feedback regulation of sex steroids has also been studied in several fishes, including spotted scat [47] and pompano [50]. However, the negative effect of E2 could be diminished by using a broad-spectrum ERα-specific antagonist, which has been previously studied and proven in spotted scat [47].

*fshβ* and *lhβ* play marked roles in the regulation of gonadal development, maturation, and spawning in fish. *fshβ* initiated and controlled the gametogenesis of both male and female fish at the early stage of gametogenesis. However, the levels of *lhβ* increased in both sexes at the advanced stages of gametogenesis, stimulating the secretion of sex steroids, and initiating the final gonadal maturation [61]. The present study explained the expression pattern of pituitary GtHs and gonadal and plasma sex steroids, which exhibited significantly lower levels of GtHs and sex steroids at the ripen stage in female SYC compared to males during the gonadal developmental stages. Furthermore, mRNA expression levels of pituitary GtHs, especially *lhβ* subunits, did not increase at the ripen stage compared to the developing stage in females. Similarly, E2 production in the ovary and serum was not significantly changed between the developing and ripen stage in females. These results might facilitate the process of reproductive dysfunction in female SYC during their final maturation. Low levels of the GtH subunits, especially *lhβ*, cause reproductive dysfunction in captive-reared female chub mackerel, which similarly failed to undergo complete final maturation [9]. Low levels of GtHs and sex steroids are also reported in captive-reared females compared to male and wild females in several fishes, including jack mackerel [8], yellowtail tetra [22], mahseer [23], mahi-mahi [24], and sablefish [25], which leads to reproductive dysfunction in captive-reared females of particular fish species.

## 4. Materials and Methods

### 4.1. Experimental Fish and Husbandry

Adult SYC at the reproductive regression stage were collected from the “grow-out” tank of the Ocean and Fisheries Science Institute, Yeonggwang-gun, Jeollanam-do, Republic of Korea. The SYC were raised in the same institute. All collected fish were reared in a conditioning tank with a continuous flow of seawater and aeration. Fish were maintained under natural daylight conditions. After conditioning for two weeks, the fish were shifted to a broodstock-rearing tank. The water temperature of the broodstock tank was initially at natural temperature (11 °C) and then it was gradually increased to 18 °C (1 °C at each 7-day interval) and held at 18 °C until breeding commenced. Fish were fed with small shrimp and supplemented with pellet food twice per day. The flow rate of the seawater in the broodstock tank was maintained at 1.5 L/min, and a continuous oxygen supply was maintained.

### 4.2. Sample Collection from Different Experimental Conditions

Various experimental adult SYC samples, for both sexes, were taken from the broodstock tank and transported to the Laboratory of Molecular Physiology, Department of Fisheries Science, Chonnam National University, Republic of Korea. Prior to tissue collection, fish samples were anesthetized using tricaine methanesulfonate (MS222). The total lengths of all fish were measured using a measuring scale, and the total weights of all fish were taken using an electronic balance. The mean body length and body weight were 25.12 ± 8.64 cm and 90.29 ± 19.13 g, respectively. Pituitary, gonad, and blood samples were collected from different experimental SYC.

- Sampling of blood: Blood samples (1–2 mL) were drawn from the caudal vein of all sampled fish (10 fish at each developmental stage) using a 5 mL syringe and were maintained in heparinized tubes. Centrifugation was performed at 5000× *g* for 15 min at 4 °C to separate the plasma. Plasma was transferred to fresh tubes and preserved at −80 °C until the extraction of steroids.
- Sampling of the pituitary: The pituitaries of each sampled fish were collected carefully from underneath the brain by dissecting the head of the fish. Afterward, they were washed with 1× PBS and immediately frozen in liquid nitrogen. Frozen samples were kept at −80 °C until the total RNA was extracted.
- Sampling of gonads: Gonad samples were collected by dissecting the belly of the fish, washed with 1× PBS, flash frozen in liquid nitrogen, and stored at −80 °C until total RNA extraction. Furthermore, a portion of the gonad was fixed in 4% PFA for gonadal histology.

#### 4.2.1. Different Organ Tissues from Captive-Reared Mature SYC of Both Sexes

Different organ tissues, such as the brain (BRN), pituitary (PIT), muscle (MUS), heart (HRT), liver (LIV), gill (GIL), kidney (KID), intestine (INT), and gonad (GND), were collected from 10 captive-reared mature fish of both sexes.

#### 4.2.2. Samples from Different Gonadal Developmental Stages of Captive-Reared SYC

Both sexes of SYC were taken from the broodstock conditioning tank at different gonadal developmental stages. Gonadal developmental stages of SYC were verified and confirmed by gonadal histology, as described previously [35]. Developmental stages included the immature (IM), developing stage (DS), ripen stage (RS), and spent stage (SS). Ten fish of each sex were sampled on each sampling day. After anesthetization, blood, pituitaries, and gonads were collected and stored.

#### 4.2.3. Pituitary Samples from In Vivo Induction of GnRHa in SYC

Samples (blood, pituitary, and gonad) were collected from both sexes of SYC, following in vivo administration of GnRHa (Sigma, St. Louis, MO, USA), as described previously [32,47,50], with a few modifications. Briefly, GnRHa was dissolved in distilled water and a working solution was prepared using 1× PBS. Both sexes of SYC at the ripen stage were divided into four groups. Three groups of SYC were injected intramuscularly with GnRHa, at concentrations of 10, 50, or 100 ng/100 g-bw (gram/bodyweight). In each treatment group, 15 SYC of each sex were injected. In the control group, 1× PBS was injected intramuscularly. Next, the pituitary samples from the male and female SYC were collected at 6 h, 12 h, and 24 h post-injection.

#### 4.2.4. Samples from Different Induced Spawning Events of Captive-Reared SYC

Blood, pituitary, and gonad samples were collected from both sexes of SYC during the different induced spawning events. Fully ripen SYC, of both sexes, were induced by administering GnRHa (Sigma, St. Louis, MO, USA), at a dose of 100 ng/100 g-bw in females and 10 ng/100 g-bw in males, for final gonadal maturation. After the administration of GnRHa, male and female fish were kept in a circular spawning tank. A continuous flow of seawater and oxygen was maintained. Spawning occurred approximately 24 h after induction. Tissue sampling was performed at different stages, such as before spawning, or just before induction of GnRHa (BSW), after 24 h of GnRHa administration, or during the release of gametes or spawning (DSW), and post-spawning (PSW). The PSW samples were collected after 7 days of spawning. Samples were collected from 10 fish for each sex and at each sampling point. After anesthetization, blood, pituitaries, and gonads were collected and stored.

#### 4.2.5. Pituitary Samples from In Vitro Cultures with E2 and MT

Pituitaries of SYC from both sexes were cultured in vitro with E2 or MT, as described previously [32,47,50]. Briefly, stock solutions of E2 and MT (Sigma, St. Louis, MO, USA) were prepared using acetone and absolute ethanol, respectively. A working solution for E2 and MT was prepared using 1× PBS. Pituitaries from 80 mature SYC, for each sex, were collected and washed with M199 medium modified with Hanks’ balanced salts (Gibco, Grand Island, NY, USA). Then, the pituitaries were placed in the holes of 12-well culture plates in 3 treatment groups and a control group. Ten pituitaries were placed in each well and preincubated at 25 °C for 2 h in serum-free M199 medium (modified with Hanks’ balanced salts), treated with penicillin–streptomycin. After 2 h of incubation, the M199 medium was disposed of, and the pituitaries were washed twice with the M199 medium. Then, fresh M199 medium (modified with Hanks’ balanced salts) with 1, 5, or 10 μM of E2 or MT was added into the wells for the three treatment groups. In the control group, only M199 medium was added. Then, the pituitaries were incubated for 24 h. During incubation, pituitaries were collected at 6 h, 12 h, and 24 h, washed with fresh 1× PBS, and preserved at −80 °C, until tRNA extraction.

### 4.3. Gonadal Histology

#### 4.3.1. Sample Preparation for Frozen Section

Gonad samples for frozen sectioning were prepared as described previously [62], with slight modification. Briefly, gonad samples were fixed in 4% PFA for overnight. After fixation, samples were washed with 1× PBS thrice and infiltrated with 15% sucrose and later with 30% sucrose. Infiltrated samples were then embedded in optimal cutting temperature (OCT) compound (Surgipath FSC 22 Clear, Leica, Richmond, IL, USA) and frozen at −20 °C overnight. The embedded tissues were then cryo-sectioned at 7–8 µm thickness using a cryostat microtome (Leica CM3050; Wetzlar, Germany) and fixed on Fisherbrand SuperFrost^TM^ Plus slides (Fisher Scientific, Pittsburgh, PA, USA). Slides were then stored at −20 °C until stained with hematoxylin and eosin (H&E).

#### 4.3.2. H&E Staining

Frozen sections were stained with Mayer’s hematoxylin and counter stained with eosin Y (alcoholic) using a H&E staining kit (ab245880; Abcam, Boston, MA, USA) as described previously [63] and according to the kit protocol. Stained gonadal tissue sections were subsequently observed under a microscope (Eclipse E600, Nikon, Tokyo, Japan) to confirm gonadal developmental stages.

### 4.4. Quantitative Real-Time PCR (qRT-PCR)

#### 4.4.1. RNA Extraction and cDNA Syntheses

Total cellular RNA (tRNA) was extracted from all sampled tissues using an ISOSPIN Cell and Tissue RNA kit (Nippon Gene, Tokyo, Japan). Quality and quantity of tRNA were evaluated by electrophoresis and spectrophotometry (ASP 2680 spectrophotometer, ACTGene, Piscataway, NJ, USA). First-stand cDNA was synthesized from 4 μL of total RNA using the oligo(dT) primer and a Superscript III First-strand cDNA synthesis kit (Invitrogen, Waltham, MA, USA). RNA extraction and cDNA synthesis were performed following the manufacturer’s protocol.

#### 4.4.2. SYC GtHs Sequence Search and Primer Design

Nucleotide sequences for the *fshβ*, *lhβ*, and *gpα* genes in the SYC were obtained from the NCBI nucleotide database search. Detailed sequence information is presented in Table 1. Furthermore, the *β-actin* gene in the SYC was obtained from the NCBI database with the following accession number: MT330378. Gene-specific forward and reverse primers were designed from the coding regions of each gene sequence, and had a GC content of 50–58% and a melting temperature (Tm) of 58.4–60.5 °C. Primer information is presented in Table 2.

#### 4.4.3. qRT-PCR Assay

qRT-PCR was performed to quantify relative mRNA expressions of *fshβ*, *lhβ*, and *gpα* subunits in the SYC tissue samples for each experiment. The mRNA expression levels of *fshβ*, *lhβ*, and *gpα* were analyzed in the pituitaries at various gonadal developmental stages, including pituitaries from in vivo administration with GnRHa peptide, pituitaries from different induced spawning events, and pituitaries from in vitro incubation with E2 or MT experiments.

All qRT-PCR assays were conducted following a protocol as described previously [32], using a 2× qPCRBIO SyGreen Mix Lo-Rox kit (PCR Biosystems Ltd., Wayne, PA, USA) in a LightCycler^®^ 96-Real-Time PCR System (Roche, Germany). A 20 μL reaction mixture was prepared using 1 μL cDNA template, 1 μL each of forward and reverse primer (Table 1), 10 μL of SyGreen Mix, and 7 μL of ultra-pure water. All tissue samples were performed in triplicate reactions for the target and reference genes. A set of reactions were conducted without the template cDNA for each experiment, designated here as the blank control, as described previously [38]. PCR amplification conditions were set to a preincubation step at 95 °C for 2 min, followed by 40 cycles of a 3-step amplification at 95 °C for 30 s, 60 °C for 20 s, and 72 °C for 30 s. The melting temperature was set as the instrument’s default setting. At the end of each cycle, a fluorescence reading was recorded for quantification. The relative gene expressions of *fshβ*, *lhβ*, and *gpα* for each experiment were analyzed according to the 2^−ΔΔCT^ method with the *β-actin* gene as the internal reference.

### 4.5. Enzyme-Linked Immunosorbent Assay (ELISA)

#### 4.5.1. Extraction of Steroid Hormones

Steroid hormones were extracted from plasma and gonad tissues, following the standard protocol described previously [15], with slight modifications as recommended by the ELISA kit.

- Plasma steroid extraction: First, 1 mL of the serum sample and an equal amount of diethyl ether were added to an Eppendorf and vortexed to mix. The top ether layer was carefully transferred to a new tube. This step was repeated twice, and the ether layers were combined in the tube. Then, the ether was evaporated to dryness from the sample under nitrogen. Then, the extracted steroid was resuspended in the supplied assay buffer and stored at −80 °C until use.
- Gonad tissue steroid extraction: First, gonad tissues were homogenized in 20 mM Tris–NaCl and sonicated. Then, the samples were centrifuged at 15,000× *g* for 15 min at 4 °C. Supernatant was collected and transferred to a new Eppendorf tube. Then, the steroids were extracted using diethyl ether, as described for the serum sample.

#### 4.5.2. ELISA Protocol

The concentration of each sex steroid (T, E2, P) in the serum and gonad samples were measured using commercial ELISA kits (MyBioSource, Inc., San Diego, CA, USA), which are specifically designed for the measurement of steroids in fish species. Three different ELISA kits were used to measure the amount of testosterone (Cat. No. MBS933475), E2 (Cat. No. MBS700179), and P (Cat. No. MBS706075). The assay was performed according to the manufacturer’s instructions. The optical densities of testosterone, E2, and P were measured at 405 nm using an Epoch™ microplate spectrophotometer (BioTek Instruments Inc., Winooski, VT, USA).

#### 4.5.3. ELISA Data Analysis

The average optical density of the blanks was deducted from the average values of each standard and unknown sample. Finally, steroid concentrations for each unknown sample were deduced from the standard curve of standard samples for each steroid.

### 4.6. Statistical Analysis

The mRNA expression values for the three GtH subunits in the pituitaries and the concentrations of the steroids in the serum and gonads in each different experiment were statistically analyzed and expressed as mean ± standard error of the mean (SEM). Changes to the relative mRNA expressions of the GtH subunits and the steroid concentrations in the serum and gonads for each different sample were analyzed using an ordinary one-way analysis of variance (ANOVA) in GraphPad Prism ver. 9.4.1 (GraphPad Software LLC., San Diego, CA, USA). Tukey’s multiple comparison test was performed to assess statistically significant differences among different experimental tissues. Statistical significance was set at *p* < 0.05. The mRNA expression values of the three GtH subunits were analyzed, in a dose- and time-related manner for GnRHa in vivo and E2 orMT in vitro, using an ordinary one-way ANOVA, while the statistical significance for each was compared to the zero time point of the initial control (IC). All graphs were prepared in GraphPad Prism 9.4.1 software. In all graphs, raw data points (circles) represent biological replicates, error bars represent SEM, and different letters or asterisks indicate significant differences (*p* < 0.05).

## 5. Conclusions

Overall, this study has evaluated the key information on the potential role of GtHs and sex steroids in captive-reared SYC. Here, *fshβ*, *lhβ*, and *gpα* were predominantly expressed in the pituitary of the SYC. The levels of pituitary GtHs and gonadal and plasma sex steroids were upregulated in male SYC during RS and at spawning time. However, the expression of GtHs and gonadal and plasma sex steroids did not show any significant changes in captive-reared female SYC. Furthermore, in vivo administration of GnRHa upregulated the expression of *fshb*, *lhβ*, and *gpα* subunits in a dose- and time-related manner. The in vitro study on the feedback regulation of sex steroids revealed that E2 and MT could arbitrate negative feedback on *lhβ* expression in female SYC. Cumulatively, it can be concluded that GtHs play a critical role in the regulation of gonadal maturation, while the sex steroids analyzed in this study might possess a negative feedback role on *lhβ* in female SYC. Finally, there were lower levels of the pituitary GtH subunits, particularly *lhβ*, and the sex steroid E2 in females compared to males, which might indicate a potential reason of reproductive dysfunction in captive-reared female SYC.

## Figures and Tables

**Table 1:** Information of SYC *fshβ*, *lhβ*, and *gpα* gene sequences and identities of FSHβ, LHβ, and GPα amino acids between SYC and other related fishes.

	Gonadotropin Subunits		
	*fshβ*	*lhβ*	*gpα*
*Sequence Information*			
NCBI nucleotide accession number	MT239441	MW192797	MW192796
NCBI protein accession number	QNG62443	QOW40925	QOW40924
ORF (bp)	348	444	399
Amino acid sequence (aa)	115	147	132
*Amino acid sequence identity (%)*			
Large yellow croaker	95.01	95.24	93.18
Largemouth bass	75.65	84.25	85.61
Skipjack tuna	72.55	92.11	90.32
European seabass	71.30	85.03	84.68
Red seabream	66.09	81.51	88.03
Chub mackerel	66.09	80.95	84.62
Pike perch	60.00	80.88	73.48

**Table 2:** qRT-PCR primer information designed from SYC GtH subunits and β-actin genes.

Name	Nucleotide Sequence (5′–3′)	GC Percentage	Tm (°C)	Length (bp)	Accession No.
SYC-FSHβ-Fw	GGCAACACCGAGTTCATCG	58	59.5	199	MT239441
SYC-FSHβ-Rv	CGTTGCATGCGCTACACTC	58	59.5		
SYC-LHβ-Fw	CATCACCAAGGACCCTGTC	58	59.5	198	MW192797
SYC-LHβ-Rv	CTCTCGAAGGTGCTGTCAG	58	59.5		
SYC-GPα-Fw	TGCACACTGAGCAAGAACAG	50	58.4	178	MW192796
SYC-GPα-Rv	CCTCTATCTCGTAGCTGTGC	55	60.5		
SYC-β-Actin-Fw	GCTGTCTTCCCATCCATCG	58	59.5	193	MT330378
SYC-β-Actin-Rv	CGTTGTAGAAGGTGTGATGC	50	58.4		

## Data Availability

Not applicable.

## Supplementary Materials

The supporting information can be downloaded at: https://www.mdpi.com/article/10.3390/ijms24108919/s1.
