# Supplementary material for: Gonadotropins and Sex Steroid Hormones in Captive-Reared Small Yellow Croaker (Larimichthys polyactis) and Their Role in Female Reproductive Dysfunction
Source: Int J Mol Sci. 2023 May 17;24(10):8919. doi: 10.3390/ijms24108919 (PMC10219107; doi:10.3390/ijms24108919)
Supplement: Supplementary file 1 [file ijms-24-08919-s001.zip › ijms-2340158-supplementary.pdf]

Gonadotropins and Sex Steroid Hormones in Captive-Reared Small Yellow Croaker and Their Role in Female Reproductive Dysfunction

Supplementary Table S1. Relative mRNA expression levels of *fshβ*, *lhβ* and *gpα* subunits in in different organs of captive-reared mature male and female small yellow croakers. Different superscript letters indicate significant differences (p < 0.05) among organs. Significant differences were calculated separately for FSHβ, LHβ, and GPα subunit. BRN: brain; PIT: pituitary; GIL: gill; LIV: liver; HRT: heart; KID: kidney; MUS: muscle; GND: gonad.

Gene	Sex	Organs								
		BRN	PIT	GIL	LIV	HRT	KID	MUS	INT	GND
fshβ	Male	0.73±0.02^{d}	4.97±0.20^{a}	0.03±0.00^{e}	0.06±0.01^{e}	0.03±0.00^{e}	0.03±0.00^{e}	0.02±0.00^{e}	0.03±0.00^{e}	1.61±0.08^{c}
	Female	0.62±0.03^{d}	3.61±0.30^{b}	0.03±0.00^{e}	0.08±0.00^{e}	0.07±0.01^{e}	0.04±0.01^{e}	0.02±0.00^{e}	0.03±0.00^{e}	1.19±0.11^{d}
lhβ	Male	1.12±0.05^{d}	5.81±0.32^{a}	0.09±0.01^{f}	0.07±0.01^{f}	0.05±0.00^{f}	0.03±0.00^{f}	0.04±0.00^{f}	0.03±0.00^{f}	1.89±0.19^{c}
	Female	0.68±0.04^{e}	3.34±0.19^{b}	0.11±0.01^{f}	0.09±0.00^{f}	0.06±0.01^{f}	0.05±0.00^{f}	0.04±0.00^{f}	0.03±0.01^{f}	1.07±0.18^{d}
gpα	Male	0.99±0.07^{c}	4.12±0.31^{a}	0.20±0.04^{e}	0.09±0.00^{e}	0.11±0.01^{e}	0.12±0.0^{e}	0.05±0.00^{e}	0.10±0.02^{e}	0.56±0.03^{c}
	Female	0.64±0.04^{c}	3.16±0.16^{b}	0.08±0.00^{e}	0.13±0.01^{e}	0.08±0.00^{e}	0.17±0.03^{e}	0.10±0.02^{e}	0.10±0.02^{e}	0.48±0.08^{d}

Supplementary Table S2. Relative mRNA expression levels of *fshβ*, *lhβ* and *gpα* subunits in the pituitary during gonadal developmental stages in male and female captive-reared small yellow croakers. Different superscript letters indicate significant differences (p < 0.05) among developmental stages. Significant differences were calculated separately for FSHβ, LHβ, and GPα subunit. IM: immature; DS: developing stage; RS: ripen stage; SS: spent stage.

Gene	Sex	Developmental Stages			
		IM	DS	RS	SS
fshβ	Male	2.16±0.19^{c}	3.86±0.28^{b}	5.19±0.40^{a}	2.56±0.15^{c}
	Female	1.94±0.13^{c}	2.84±0.22^{bc}	3.53±0.20^{b}	3.09±0.14^{bc}
lhβ	Male	2.05±0.01^{c}	3.36±0.22^{b}	5.90±0.34^{a}	2.15±0.29^{c}
	Female	1.93±0.28^{c}	2.91±0.38^{bc}	4.47±0.34^{b}	2.18±0.11^{bc}
gpα	Male	1.54±0.04^{d}	2.41±0.25^{bc}	3.92±0.12^{a}	1.78±0.22^{cd}
	Female	1.72±0.04^{cd}	2.49±0.16^{bc}	3.30±0.17^{b}	1.97±0.03^{cd}

Supplementary Table S3. Relative mRNA expression levels of *fshβ*, *lhβ* and *gpα* subunits in the pituitary during induced spawning events in captive-reared male and female small yellow croaker. Different superscript letters indicate significant differences ($p < 0.05$) among different spawning events. Significant differences were calculated separately for FSHβ, LHβ, and GPα subunit. BSW: before spawning; DSW: during spawning; PSW: post-spawning.

Gene	Sex	Induced Spawning Events		
		BSW	DSW	PSW
fshβ	Male	5.34±0.49^{c}	8.40±0.62^{a}	1.89±0.05^{d}
	Female	3.67±0.23^{c}	7.16±0.30^{b}	4.29±0.15^{c}
lhβ	Male	6.01±0.12^{c}	12.84±0.65^{a}	1.92±0.06^{d}
	Female	4.66±0.30^{c}	10.03±0.83^{b}	5.33±0.54^{c}
gpα	Male	5.33±0.25^{b}	7.85±0.22^{a}	2.00±0.17^{d}
	Female	3.78±0.39^{c}	6.91±0.40^{a}	3.29±0.29^{c}

Supplementary Table S4. Concentrations of estradiol (E2), testosterone (T), and progesterone (P) in the gonad of captive-reared male and female small yellow croakers during gonadal developmental stages. Different superscript letters indicate significant differences ($p < 0.05$) among developmental stages. Significant differences were calculated separately for E2, T, and P. IM: immature; DS: developing stage; RS: ripen stage; SS: spent stage.

Steroids	Sex	Developmental Stages			
		IM	DS	RS	SS
Estradiol (E2) (pg/ml)	Male	$425±9.54^{d}$	$521±7.64^{b}$	$651±6.89^{a}$	$406±4.91^{d}$
	Female	$226±4.33^{f}$	$349±8.74^{e}$	$469±4.73^{c}$	$332±7.22^{e}$
Testosterone (T) (ng/ml)	Male	$6.12±0.10^{f}$	$10.62±0.10^{c}$	$14.56±0.08^{a}$	$6.90±0.07^{e}$
	Female	$4.19±0.04^{g}$	$7.74±0.07^{d}$	$11.15±0.07^{b}$	$4.06±0.04^{g}$
Progesterone (P) (ng/ml)	Male	$12.44±0.22^{f}$	$16.76±0.16^{d}$	$24.03±0.22^{a}$	$17.37±0.07^{c}$
	Female	$10.09±0.21^{g}$	$14.36±0.33^{e}$	$20.92±0.46^{b}$	$12.05±0.30^{f}$

Supplementary Table S5. Concentrations of estradiol (E2), testosterone (T), and progesterone (P) in the serum of captive-reared male and female small yellow croakers during gonadal developmental stages. Different superscript letters indicate significant differences ($p < 0.05$) among developmental stages. Significant differences were calculated separately for E2, T, and P. IM: immature; DS: developing stage; RS: ripen stage; SS: spent stage.

Steroids	Sex	Developmental Stages			
		IM	DS	RS	SS
Estradiol (E2) (pg/ml)	Male	$133±6.25^{c}$	$203.7±3.84^{b}$	$261±9.02^{a}$	$106.7±5.81^{c}$
	Female	$105±3.79^{c}$	$187.7±5.24^{b}$	$203.3±5.04^{b}$	$114.3±7.27^{c}$
Testosterone (T) (ng/ml)	Male	$3.29±0.10^{d}$	$4.29±0.14^{b}$	$5.14±0.06^{a}$	$3.47±0.04^{d}$
	Female	$2.84±0.19^{f}$	$2.99±0.06^{e}$	$3.81±0.17^{c}$	$2.61±0.14^{f}$
Progesterone (P) (ng/ml)	Male	$5.95±0.09^{d}$	$8.61±0.19^{b}$	$13.52±0.18^{a}$	$7.42±0.24^{c}$
	Female	$4.48±0.16^{e}$	$6.96±0.12^{c}$	$8.90±0.09^{b}$	$5.53±0.17^{d}$
